# Centering Patient Expectations of a Novel Home-Based Oral Drug Treatment among *T. b. rhodesiense* Human African Trypanosomiasis Patients in Uganda

**DOI:** 10.3390/tropicalmed5010016

**Published:** 2020-01-21

**Authors:** Shona J Lee, Renah J Apio, Jennifer J Palmer

**Affiliations:** 1 Department of Global Health & Development, London School of Hygiene and Tropical Medicine, Keppel Street, London WC1H 9SH, UK; jennifer.palmer@lshtm.ac.uk; 2 Dokolo Health Centre, Dokolo, Uganda; apiojanetrenah9@gmail.com; 3 Centre of African Studies, University of Edinburgh, 15a George Square, Edinburgh EH8 9LD, UK

**Keywords:** human Africa trypanosomiasis, fexinidazole, home-based treatment, patient-centred care, Uganda, elimination

## Abstract

The recent approval of fexinidazole for human African trypanosomiasis (HAT) caused by *T. b. gambiense* enables improved patient management that is pivotal to elimination. Effective in both the early and late stages of the disease, it obviates the need for invasive lumbar punctures which guide therapy, in some patients. Unlike existing injectable treatments requiring systematic hospitalisation, fexinidazole’s oral administration will allow many patients to be treated in an outpatient or home-based setting. Drawing on interviews with 25 *T. b. rhodesiense* HAT patients managed under existing protocols in Uganda where trials of fexinidazole will begin shortly, this article explores patient expectations of the new protocol to help HAT programmes anticipate patient concerns. Alongside frightening symptoms of this life-threatening illness, the pain and anxiety associated with lumbar punctures and intravenous injections of melarsoprol contributed to a perception of HAT as a serious illness requiring expert medical care. While preferring a new protocol that would avoid these uncomfortable procedures, patients’ trust in the care they received meant that nearly half were hesitant towards shifting care out of the hospital setting. Clinical observation is an important aspect of existing HAT care for patients. Programmes may need to offer extensive counselling and monitoring support before patients are comfortable accepting care outside of hospitals.

## 1. Introduction

The recent regulatory approval of fexinidazole for the treatment of human African Trypanosomiasis (HAT, also known as sleeping sickness) caused by the most common, *T. b. gambiense* form, has been hailed as a “big step toward the gradual elimination of this disease from sub-Saharan Africa” [1]. The World Health Organisation (WHO) added fexinidazole to the Essential Medicines List for treatment of *gambiense* HAT, following successful clinical trials in the Democratic Republic of Congo (DRC) and Central African Republic [2]. Apart from the adoption of a shorter and safer dosing schedule for melarsoprol in 2010 [3,4], patients suffering from *T. b. rhodesiense* HAT have benefitted little from wider efforts to reform HAT treatment since the early 1980s [5,6,7,8,9,10,11]. Trials to test fexinidazole’s efficacy against *rhodesiense* HAT, however, are also currently underway in Malawi and about to begin in Uganda [12]. Fexinidazole, a nitroimidazole treatment that can be taken orally once a day for 10 days [11,12,13], in many ways simplifies HAT treatment regimens, enabling new forms of patient management.

One simplification relates to the use of invasive lumbar punctures. Until recently, HAT programmes have had to balance the risks of administering drugs that were either ineffective or too toxic. Melarsoprol, for example, the only drug effective against the second meningo-encephalitic stage of *rhodesiense* HAT, is notoriously painful to administer and can cause post-treatment reactive encephalopathy, contributing to a treatment-associated fatality rate of about 6% [13,14]. Suramin, a safer drug, can only be used for the first haemo-lymphatic stage because it cannot penetrate the blood–brain barrier. Likewise, in *gambiense* HAT, the safer but less effective drug, pentamidine, is used in the first stage while nifurtimox-eflornithine combination therapy (NECT) is reserved for the second stage. Lumbar punctures are routinely used to determine whether parasites are present in the cerebrospinal fluid to guide HAT treatment decisions.

Capable of penetrating the blood–brain barrier, however, fexinidazole is safe and effective in both the first and second stages of *gambiense* disease [15]. NECT is still recommended as a first-line treatment for patients in “severe second stage” [16] (p.7), defined immunologically as corresponding to a white blood cell count of ≥100/μL of cerebrospinal fluid. Recent (‘interim’) changes to the WHO HAT treatment guidance, however, empower clinicians to use their clinical judgement in categorizing disease severity by evaluating patients’ presenting symptoms. This guidance advises them to perform lumbar punctures only when severe disease is suspected. The use of fexinidazole as a first-line treatment for the first and early second stage of the disease therefore obviates the need for invasive lumbar punctures in many patients.

Another simplification relates to hospitalisation. Unlike existing treatments, which involve labour-intensive intravenous infusions and carry a risk of catheter or needle-related infection [16], fexinidazole’s oral administration route is also believed to be safe enough not to require systematic hospitalisation. A subset of patients who are deemed sufficiently healthy and have a supportive caregiving network could be cared for in an outpatient or home-based setting. While the WHO’s interim guidelines currently recommend home-based treatment using fexinidazole only if its administration is supervised directly by a healthcare worker [16], home-based treatment administered by caregivers is currently being trialled in a Phase 3b implementation study in the DRC [17].

Toxic medicines, lumbar punctures, and long-term hospitalisation have been hallmark, negative experiences of HAT patient care since the early 20^th^ century [18,19,20], and collective memories of past control campaigns influence present day HAT awareness and perceptions of risk [21,22,23]. In the colonial period, lumbar punctures were sometimes administered with violence and patients were forced to stay in treatment camps for years [19]. In the contemporary context, fear of lumbar punctures has been implicated in patients’ reasons not to attend mass screening campaigns for HAT or complete diagnostic referrals from peripheral facilities [24,25], and the financial costs of hospitalisation are thought to dissuade patients from completing treatment because of the need to pay for transportation, medical supplies, and food while also dealing with interrupted employment [26].

While a new patient management protocol that circumvents lumbar punctures, more toxic alternatives, and systematic hospitalisation would presumably be welcomed by patients and healthcare workers in endemic settings, these peoples’ receptivity towards such technological changes remains unexplored. Introducing novel technologies and strategies into an established ecosystem of care can have unintended effects on the socio-technical relationships that have been formed around HAT case management [25]. Compliance with a new treatment protocol, particularly in a home-based setting, is likely to be contingent not only on patients’ trust in the new drug, but also on the institutions and people introducing them [25,27]. Moreover, studies on the extension of hospital care into the home have demonstrated how experiences with older interventions can ‘resurface’ in contemporary programmes through the “historical longevity of similar groupings of practices and ideas” [28] (p.156), highlighting the importance of examining patient receptivity towards home-based treatments in relation to past and present associations with hospital-based care.

Drawing on interviews with *T. b. rhodesiense* HAT patients managed under existing protocols at two regional treatment centres in Uganda where trials of fexinidazole will begin shortly, this article explores patient receptivity towards key elements of the new fexinidazole patient management protocol. Reflecting on their experiences of hospital treatment and the prospect of an alternative future of HAT care, it describes, from a patient perspective, the existing context of care into which fexinidazole will be introduced. In so doing, we hope to help HAT elimination programmes anticipate key patient concerns and find ways that they can introduce fexinidazole that are culturally appropriate and relevant [29].

## 2. Materials and Methods

### 2.1. Context

In Uganda, cases of *T. b. rhodesiense* HAT have declined from 1417 reported in 1990 to only 4 in 2018 [14,30,31,32]. This study took place between January and March 2016 in Lwala and Dokolo treatment centres in northern Uganda, which manage HAT cases from the historically endemic districts of Dokolo, Kaberamaido, Apac, and Lira. These are now the sites of a clinical trial that will compare the efficacy and safety of fexinidazole to melarsoprol in *T. b. rhodesiense* patients led by the national HAT control programme in partnership with a global product development partnership organisation, the Drugs for Neglected Diseases Initiative (DNDi) under the HAT-r-ACC consortium [32,33].

At the time of fieldwork, suramin and melarsoprol were the treatments used for first and second stage HAT, respectively, and were given according to the WHO’s guidelines [34]. Lumbar punctures were systematically done to guide treatment decisions. Suramin was administered intravenously in five weekly doses and melarsoprol in 10 daily doses alongside a 12-day course of prednisolone. Most patients were therefore hospitalized for between 2 and 6 weeks.

### 2.2. Study Design

*T. b. rhodesiense* HAT patients were interviewed to document their experiences of HAT and the HAT control programme, including their experiences of treatment-seeking, diagnosis, disease staging, treatment, and post-treatment follow-up. At the time, fexinidazole was undergoing phase 3 trials in the DRC. With such a transformative treatment on the horizon, patients and healthcare workers were invited to reflect on their experiences of the diagnostic staging process and treatment to explore their hopes and concerns about an alternative oral treatment that could be given on the basis of positive microscopy, in some cases without requiring lumbar puncture, and which could potentially be taken at home. Healthcare workers interviewed as part of a related study on HAT surveillance capacity in the region were similarly invited to share their views on the introduction of these prospective treatment changes and were included in this analysis.

The study protocol was reviewed and approved by research ethics committees at the University of Edinburgh, Busitema University, and the Ugandan National Council for Science and Technology (UNCST, approval code HS 1876).

### 2.3. Patient Sample Selection

As only one patient was undergoing treatment at the time of the study, we relied mainly on the accounts of patients who had been treated and discharged within the prior 26 months. The interview sample (25 patients) was drawn from this overall pool (100 patients treated between 01/01/2014 and 28/02/2016, Table 1) and was selected with the assistance of clinical staff responsible for HAT case management at the two treatment centres.

To collect a wide range of potentially positive and negative treatment experiences, the sampling strategy focused on recruiting a similar number of patients who (a) had returned to the hospital at least once after treatment for a quarterly follow-up appointment or not; (b) were in different disease stages; and (c) had been treated at each facility. One patient was undergoing treatment at the time of the study, so was interviewed there; 22 others were selected and successfully traced at home and invited for interview. Two further respondents who had been treated a decade previously at Lwala (one in 2005, and another in 2004 and again in 2006 after a second infection/relapse) were also invited to interview at the suggestion of other patients or staff to provide a long-term perspective.

Thirteen out of the 25 (52%) patients interviewed were male. The median age of our sample was 30 (range 12–80), which was slightly older than the wider pool of patients. Seventeen patients (68%) were in second stage and were treated with melarsoprol. Three patients relapsed after treatment failures for first stage infections and were later readmitted for second stage treatment. At the time of study, thirteen patients (52%) had attended a follow-up appointment.

### 2.4. Recruitment and Interviews

Participants were mobilised for interviews at least 2 days prior by telephone and/or in person through village health team workers who had been briefed on the research and could explain the study to them in their own language. For patients under 18 years of age at the time of interview, adult guardians were interviewed instead. Verbal informed consent was obtained from patients on initial contact and written (or witnessed oral consent with the provision of a thumbprint) was recorded before the interview. All those invited into the study consented to participate. Interviews took place at the patient’s home or a local health centre per their request. Interviews followed a semi-structured interview guide and took place through consecutive translations to local languages by trained interpreters, as necessary. Discussions were audio-recorded and transcripts were produced shortly afterwards.

### 2.5. Analysis

Each participant was attributed a unique identification code with accompanying demographic characteristics to aid interpretation. Recurrent themes for each topic were then identified and coded using NVIVO software (version 11), and quotes that articulated these themes were selected to summarise key points. Theoretical concepts from the fields of medical anthropology and social studies of science and innovation were drawn on to understand the social and therapeutic landscape in which HAT medicines were introduced, accessed, and taken up in local context [20,21,22,28,29,35,36,37,38,39,40,41,42,43]. This is important in an elimination setting where treatment adherence and outcomes are essential to programme success. We present our findings beginning with the patients’ experiences of HAT symptoms, the decision-making that led them to the treatment centre, and how long periods of hospitalisation impacted their lives. We then present patient experiences of suramin and melarsoprol intravenous injections, including the lumbar puncture procedure that guided treatment decisions, alongside accounts of the attentive care of health professionals. We then describe how these experiences informed patients’ perceptions of an alternative oral treatment protocol, followed by expectations and concerns about how such a treatment could be delivered in the home setting.

## 3. Results

### 3.1. Experiences of Illness and Hospitalisation

Patients described the reputation of HAT as being a “terrible” disease that is widely feared and sometimes attributed to witchcraft; descriptions of this fear are often extended to its medications as well.

*The people here, the feeling is they fear the disease; [they think] that [it] is so terrible and at times they feel it is caused by some traditional behaviours like witchcraft. The people fear the disease and the medications. People have been tested, but apart from me, no one has been caught with the disease, but they fear it […]. It is even worse than HIV.*

—Patient 8, Dokolo

The symptoms that patients used to illustrate how terrible HAT feels included headaches, fever, an excessive need to sleep, muscle weakness or ‘paralysis’, and difficulty walking, as well as cognitive impairment.

*He had a fever, and headache, general body pain, then the muscles on the right side in his calf became so painful that it couldn’t allow him to walk. Then eventually he was behaving like someone with mental problems, like he was mentally confused.*

—Parent of Patient 2, Dokolo

*He had a headache for so long. He would be looking at someone from a distance as if there are two people [as in a hallucination]. The boy complained he would be seeing very big animals with red eyes. And he was talking in an un-coordinated manner.*

—Parent of Patient 9, Lwala

Typical of *rhodesiense* HAT infections, some people’s symptoms appeared to progressed very rapidly with escalating severity, which caused great alarm.

*I started feeling the headache—it moved from one side to the other […] then by late [evening] I started feeling a terrible fever. I became so weak I requested the children to light a fire in my house so I could go and rest. By 10pm, the thing worsened, and it started affecting all the joints, all my joints were paining me. I lost consciousness by that time […] I even went and called the catechist to come and pray for me, I thought I was on my way [going to die].*

—Patient 18, Dokolo

In many cases, previous knowledge of HAT services and high levels of institutional trust in HAT centres were important in ensuring patients sought treatment at hospitals for their symptoms, especially when this involved overriding the advice of others to seek advice from spiritual healers.

*For example, when I was taking him and he was mentally confused, someone said “is that the sort of thing you take to be treated at the hospital? Take him to the witchdoctor and he will be out with it”. But I knew that others who had similar symptoms were taken to a health centre, and from there they were treated and improved.*

—Parent of patient 2, Dokolo

Almost all patients described difficulty in reaching a hospital for admission. Whether they were brought in an incapacitated state by others or presented themselves, many faced challenges in accessing transport.

*The challenge was that I had problems in transport, the place is far and I was very weak so couldn’t get myself there. But eventually a woman rode me on a bicycle.*

—Patient 4, Dokolo

Most respondents focused heavily on the negative social and economic effects of being admitted to hospital for the duration of treatment. These included having to arrange childcare or help to look after crops, raising funds for food and medical supplies, and finding someone to support and take care of them in hospital. The socio-economic fall-out from HAT was observable in terms of educational absence, transport costs, associated medical costs, and agricultural losses.

*Other than distance, there was frequent visiting to the hospital, we had to travel there a lot, we took things like foodstuffs, it was a challenge. It affected his studies so much, he was behind in his studies.*

—Parent of patient 6, Apac

*I was treated well in terms of treatment, the problem I had was leaving home for one month—I had left rice in the garden and the daughter-in-law was struggling to harvest and had difficulties. Also raising money for the upkeep was difficult.*

—Patient 18, Dokolo

### 3.2. Receptivity Toward an Oral Treatment

A clinician working at Dokolo HC IV described how toxic and painful treatment with melarsoprol can be.

*The compound can also cause tissue necrosis, so you must be very careful to get the IV [intravenous] line right, otherwise a patient can even lose an arm […]. Suramin is just fine, there is no problem giving this, but Mel B [melarsoprol] is so, so painful for them because the drug is so viscous and thick. It’s like vegetable oil, and it needs a lot of force to push it through the vein, that is why it is so painful for them. Some say it is like having fire in their veins.*

Patients liked the idea of an oral administration format because the injections ‘burned’. Overall 23/25 (92%) patients reported they would prefer an oral drug regimen over the intravenous injection treatment they experienced (Table 2).

*The good option would be a tablet because the [melarsoprol] injection, as it is entering, it burns and needs to go just slowly.*

—Patient 1, Kaberamaido

*If side effects are not there, yes [he would prefer to take the medicine in tablet form]. The pain during [melarsoprol] injection is so painful, I would prefer getting an oral treatment that I can take from home.*

—Patient 5, Dokolo

Patient descriptions of pain from the medicine made them feel vulnerable and therefore wished to be under medical supervision, as in this comment from a patient who described falling into an incoherent state while undergoing treatment:

*For me, I appreciated that it was good for me to be there [staying in the hospital], because after I got the [melarsoprol] injection, sometimes I could not understand myself, so I think it needed me to be near [to health staff], under supervision*

—Patient 1, Kaberamaido

While the experiences described by patients of the treatment itself were unpleasant, the discomfort was nevertheless commonly acknowledged as a necessary side effect of a strong drug working in the body. Some patients, such as Patient 1 felt the oral tablet option would be ineffective as a treatment for such a serious condition, as *“the disease was so terrible that it just needed injection, the tablets could not do it.”* However, when asked what they would prefer if an oral treatment could be proven to be safe and effective, these concerns tended to wane alongside the patients’ motivation to minimise disruptions to daily activities.

*Tablets would be better if they are doing the same thing. I could continue with it [treatment] myself because I need my [social] life.*

—Patient 1, Kaberamaido

For those who could remember their diagnosis and treatment experiences in detail, the disease staging procedure was recalled as being particularly uncomfortable and debilitating. An oral treatment that could be given without the need for lumbar puncture was thus an attractive benefit of the new protocol.

*I am glad to be cleared of that disease, but I wish to no longer have that needle in my back! I could not bend for a long time, it was so painful.*

—Patient 12, Dokolo

In one case, the fear of undergoing the lumbar puncture again, combined with the apparent success of treatment, influenced the patient’s decision not to return for follow-up.

*They gave a certain period to go back, but during that time I felt so well, and also I was fearing the lumbar puncture, so I didn’t go back.*

—Patient 4, Lira

### 3.3. Receptivity Toward Home-Based Treatment Administration

When asked to consider whether they would be receptive to a home-based administration of HAT treatment, patients and their caregivers often discussed the impacts of hospitalisation on their domestic and economic lives.

*I am the one looking after him, so home is left behind with my siblings. Feeding is a problem for me for this long. At home we have our cassava garden and vegetables, but here there is no forest here for collecting firewood from. If we go back again we need transport which becomes costly […]. If there was treatment that I could carry home, then I would prefer that.*

—Relative of Patient 12, Dokolo

*[Home treatment] would be better for me, I would be doing other things at home. If I go away for over one month, when I come home it is a mess.*

—Patient 8, Dokolo

*I would prefer taking drugs at home because staying at the hospital is expensive and, even going for treatment there, we had to sell land to pay for it.*

—Patient 3, Kaberamaido

Health workers also acknowledged the struggles faced by patients during treatment and expressed hope for the benefits of a potential oral treatment that patients could take home:

*I think that would be better to manage them from home, because the society we live in and the services we have, I think our community prefer being with their people at home. Staying in hospital is an inconvenience to the patient and the family, but only if the condition can be managed from home.*

—Nurse, HCIII, Kaberamaido

Despite the substantial difficulties associated with hospitalisation uniformly expressed by patients, only half of patients 13/25 (52%) claimed that they would prefer a home-based treatment (see Table 2). The remainder expressed a preference to be treated in hospital under close observation throughout their treatment course, regardless of the route of treatment administration.

### 3.4. The Value of Clinical Monitoring

A key reason that patients expressed discomfort with receiving treatment administered at home was that they imagined their care would not be overseen by health staff. Most patients had felt well cared for when admitted to hospital for HAT treatment, as expressed in comments on the quality of care they received and the trustworthiness and capabilities of the healthcare workers who treated them.

*I believed [the diagnosis of HAT given to me] because once a medical person tells you something you cannot deny […]. I would prefer getting treatment from the hospital like before. It is important [to have] close follow-up by medical workers.*

—Patient 19, Dokolo

*There [in Lwala Hospital] it was good treatment, I would rather be at the hospital. There you get regular reviews.*

—Patient 24, Lwala

*Treatment from the hospital [is better], because it’s always close to the health workers and the observation is very close […]. I like the way they handled him at Dokolo, they paid very close attention.*

—Parent of Patient 20, Dokolo

*At hospital I was given due care perfectly.*

—Patient 16, Dokolo

*They [hospital staff] are competent, the evidence is how much I have improved, you cannot improve when you are managed by someone who is not competent.*

—Patient 8, Dokolo

In some cases, patient preferences for hospital-based administration were coupled with a concern that they might not oversee their own drug administration properly if the onus of treatment was on themselves or their families.

*I would rather be at the hospital; it [provides] good treatment because when you are there the people there are full time. There you get regular reviews, because it’s always close to the health workers and the observation is very close. At home you might forget your time and take the treatment wrong.*

—Patient 21, Dokolo

*I would prefer to be given [treatment] at the hospital by the health workers. The oral treatment to be taken, it is possible that I will forget what time and how much I should take, but the health workers at the hospital will give the right drug at the right time. I would trust the health workers.*

—Patient 4, Dokolo

Healthcare workers, too, had concerns that patients would not be confident in managing their own treatment or receive appropriate medical support at home, and proposed a model of home-based treatment administration that would be observed by community-based healthcare workers, similar to the directly observed treatment strategy (DOTS) implemented by tuberculosis control programmes in Uganda.

*I think the hospital for treatment is good because they [patients] can be constantly and professionally monitored. [The] home might not be a good environment. But if they can be observed at home, like the DOTS, it would be preferred.*

—Nurse, HCIII, Lira

*I think it is better they stay in hospital where they can be monitored. Also, if you give patients drugs […] they [the drugs] will sit there next to them in the sun. Where is there for people to store drugs properly? […]. Something like DOTs could work, I think. Although if the system of treatment were to change like that then the community would again need to be sensitised beforehand so they understand the new protocol.*

—Voluntary Health Team worker, Kobulabulu, Kaberamaido

Although the anxieties associated with painful lumbar punctures and intravenous injections shaped strong preferences for an oral treatment that could avoid such procedures, current treatment modalities, which emphasise inpatient observation, appeared to strengthen the perception of HAT as a serious illness requiring expert clinical management. In such a context, it was evident that the patients’ trust in HAT services and the staff providing it was actually stronger than their fear of HAT treatment, itself.

## 4. Discussion

Fexinidazole is among several promising new tools expected to push HAT elimination ‘to the last mile’ [27]. Acoziborole (also known as SCYX-7158), which is hypothesized to be capable of curing both stages and forms of HAT in a single oral dose, is currently undergoing Phase 2b/3 clinical trials in the DRC and Guinea [14,44], and has been described as another “game-changer” for elimination efforts [45] (p.14). Admittedly, fexinidazole may not overcome all the problems of HAT patient management. For patients with ‘severe’ second stage *gambiense* illness who respond better to NECT treatment, for example, lumbar punctures and long-term hospitalisation are likely to remain part of their experience of care, and it is unclear whether, for *rhodesiense* disease, fexinidazole will be able to replace melarsoprol or be delivered in a home-based setting.

Nevertheless, our findings suggest some clear benefits to the introduction of fexinidazole for HAT patients. Patients recalled difficult experiences of treatment and hospital admission where many struggled to pull together the money and social care required to support them through their hospitalisation. Most patients and healthcare workers in this study agreed that a course of oral tablets that could be taken at home would be convenient. Perhaps surprisingly, however, we found less enthusiasm overall for a home-based treatment option than might be expected. Nearly half of patients expressed hesitancy toward taking HAT treatment outside the hospital setting where they could not be monitored closely by medical staff.

Not only are the symptoms of HAT “terrible”, they can also be stigmatizing [23]. The frightening experiences of this life-threatening illness, alongside the pain and anxiety associated with lumbar punctures and intravenous injections (particularly for melarsoprol) contributed to the perception of HAT as a serious illness requiring expert medical care. While current treatments are uncomfortable, potentially toxic, and require long hospital stays, the specialist and attentive care they entail has helped to establish melarsoprol and suramin as trusted treatments. The hesitancy expressed by patients about a home-based treatment model were predominantly related less to the prospect of an unknown drug, however, than to satisfaction with the competence and attentiveness of the healthcare workers who cared for them under existing protocols. This can be interpreted not as resistance to a new drug regimen but rather an appreciation of existing HAT services as high-quality care that is comparatively rare in rural areas of Africa where HAT is endemic [46]. Therefore, while it is unlikely that patients with very severe symptoms would be eligible for outpatient or home-based HAT care, programmes may need to offer extensive counselling and monitoring support before even patients in early stage are comfortable accepting care in this way.

## 5. Conclusions

This study describes the contextual life-worlds that frame the treatment experiences of a sample of *T. b. rhodesiense* HAT patients in Uganda, where a safety and efficacy trial of fexinidazole will shortly begin. Despite the significant impacts that hospitalisation has on the social lives and livelihoods of *rhodesiense* HAT patients, the patient testimonies collected in this study demonstrated the high level of trust patients already have in the existing system of care and the difficulties of changing behaviours in systems more generally. 

The roll-out of fexinidazole will be a pivotal moment in the history of HAT care and control. The introduction of an oral treatment would engage with many of the legitimate needs and concerns about current treatment protocols expressed by patients and healthcare workers in this setting. These include painful lumbar puncture and injection procedures, treatment side effects, potentially fatal drug reactions, the economic costs of travel and long stays in hospital. However, meeting elimination goals will require paying close attention to structural and relational elements of health systems and cannot rely solely on the availability of diagnostics and medicines. The reluctance we observed in our study toward home-based management can be interpreted as demonstrating high existing levels of trust in the current system’s quality of care, which comprise clinical performances of monitoring and observation as much as the administration of medicines. Inpatient clinical observation appears to be an aspect of existing modes of HAT care that is important to patients. These concerns may be specific to the sample in question but nonetheless raise important considerations for devising locally specific and patient-centred approaches to introducing fexinidazole, which may also be important to patients in other settings.

Programmes introducing the fexinidazole protocol should seek to build on good relations in the existing hospital-based system of HAT treatment delivery and include flexible options for patient management procedures. This is important to ensure that HAT treatment remains patient-centred, both through technological solutions to health system challenges, but also through more relational aspects of interventions, which affect the quality of care.

## Figures and Tables

**Table 1:** Patient characteristics.

	Total	Study Site		Gender		Age (years)	Disease Stage ^1^		Attended ≥ 1 Follow-up Appointment ^2^	
		Lwala	Dokolo	Male	Female	Median (range)	First	Second	Yes	No
All admitted patients (01/01/2014 – 28/02/2016)	100	69	31	54	46	20 (2–80)	22	75	58	33
Study sample: recent treatment (2014-16)	23	11	12	12	11	30 (12–80)	7	16	11	10
Study sample: historic treatment (2004-6)	2	2	0	1	1	39.5 (29–50)	1	1	2	0

^1^ Disease stage information not available for 3 out of 100 patients admitted. ^2^ Six patients died before completing treatment and three had completed treatment within three months of the study; these patients were thus not eligible for follow-up by the programme.

**Table 2:** Patient preferences for the administration route and setting of human African trypanosomiasis (HAT) treatment.

Protocol Characteristic	Number Who Preferred Existing Protocol	Number Who Preferred Proposed Protocol
Administration route (existing: intravenous injection; proposed: oral tablets)	2 (8%)	23 (92%)
Administration setting (existing: hospital-based; proposed: home-based)	12 (48%)	13 (52%)
